# Habitability of the marine serpentinite subsurface: a case study of the Lost City hydrothermal field

**DOI:** 10.1098/rsta.2018.0429

**Published:** 2020-01-06

**Authors:** Susan Q. Lang, William J. Brazelton

**Affiliations:** 1 School of the Earth, Ocean, and Environment, University of South Carolina, Columbia, SC 29208, USA; 2 Department of Biology, University of Utah, Salt Lake City, UT 84112, USA

**Keywords:** serpentinization, deep biosphere, ocean worlds, origin of life, Lost City, Atlantis Massif

## Abstract

The Lost City hydrothermal field is a dramatic example of the biological potential of serpentinization. Microbial life is prevalent throughout the Lost City chimneys, powered by the hydrogen gas and organic molecules produced by serpentinization and its associated geochemical reactions. Microbial life in the serpentinite subsurface below the Lost City chimneys, however, is unlikely to be as dense or active. The marine serpentinite subsurface poses serious challenges for microbial activity, including low porosities, the combination of stressors of elevated temperature, high pH and a lack of bioavailable ∑CO_2_. A better understanding of the biological opportunities and challenges in serpentinizing systems would provide important insights into the total habitable volume of Earth's crust and for the potential of the origin and persistence of life in Earth's subsurface environments. Furthermore, the limitations to life in serpentinizing subsurface environments on Earth have significant implications for the habitability of subsurface environments on ocean worlds such as Europa and Enceladus. Here, we review the requirements and limitations of life in serpentinizing systems, informed by our research at the Lost City and the underwater mountain on which it resides, the Atlantis Massif.

This article is part of a discussion meeting issue ‘Serpentinite in the Earth System’.

## 1. Introduction

Serpentinites are formed when ultramafic rocks, like those in Earth's mantle, are exposed to water. The geochemical reactions that occur during serpentinization have potentially profound implications for the origins and evolution of life on Earth and other planets. Serpentinization releases hydrogen gas (H_2_) as a result of hydrating and oxidizing iron minerals, and this by-product of serpentinization is the key to its biological importance. High concentrations of H_2_ and appropriate catalysts in a hydrothermal system can lead to the abiotic synthesis of organic molecules, which provide a source of food for life and could have also played a major role in early biochemical evolution [1,2]. Serpentinites were probably more abundant and active on the early Earth than they are now [3], and serpentinization has probably occurred to some degree on all rocky planetary bodies in the solar system [3]. It may be presently active on ‘ocean worlds’ such as Europa and Enceladus [4–6]. Therefore, a better understanding of the biological opportunities and challenges in serpentinizing systems provides important insights into the potential for the origin and persistence of life on Earth and elsewhere in the solar system.

Until recently, it was accepted that all ecosystems ultimately depend on energy from the sunlight. The discovery of chemoautotrophic ecosystems in seafloor hydrothermal systems in the 1970–80s revealed that magmatic energy from the Earth's deep interior can also support robust, self-sustaining ecosystems, independently of the organic remains from photosynthesis. This discovery dramatically expanded the potential possibilities for life outside Earth to include potential habitats that are magmatically active even if they lack abundant sunlight. If it can be shown that serpentinization also supports self-sustaining ecosystems, independent of sunlight and also independent of magmatic activity, then the diversity of habitats and planets potentially capable of supporting ecosystems would be expanded even further to include any habitat that has serpentinizing rock and liquid water. However, it remains unclear whether serpentinization and its associated geochemical reactions are sufficient to support the origin and evolution of life independently of other geological processes, or if the products of serpentinization must be mixed with other materials to meet all of the requirements for continuous habitability [7]. Here, we review the potential opportunities and challenges for life in marine serpentinizing systems.

**Figure 1.**
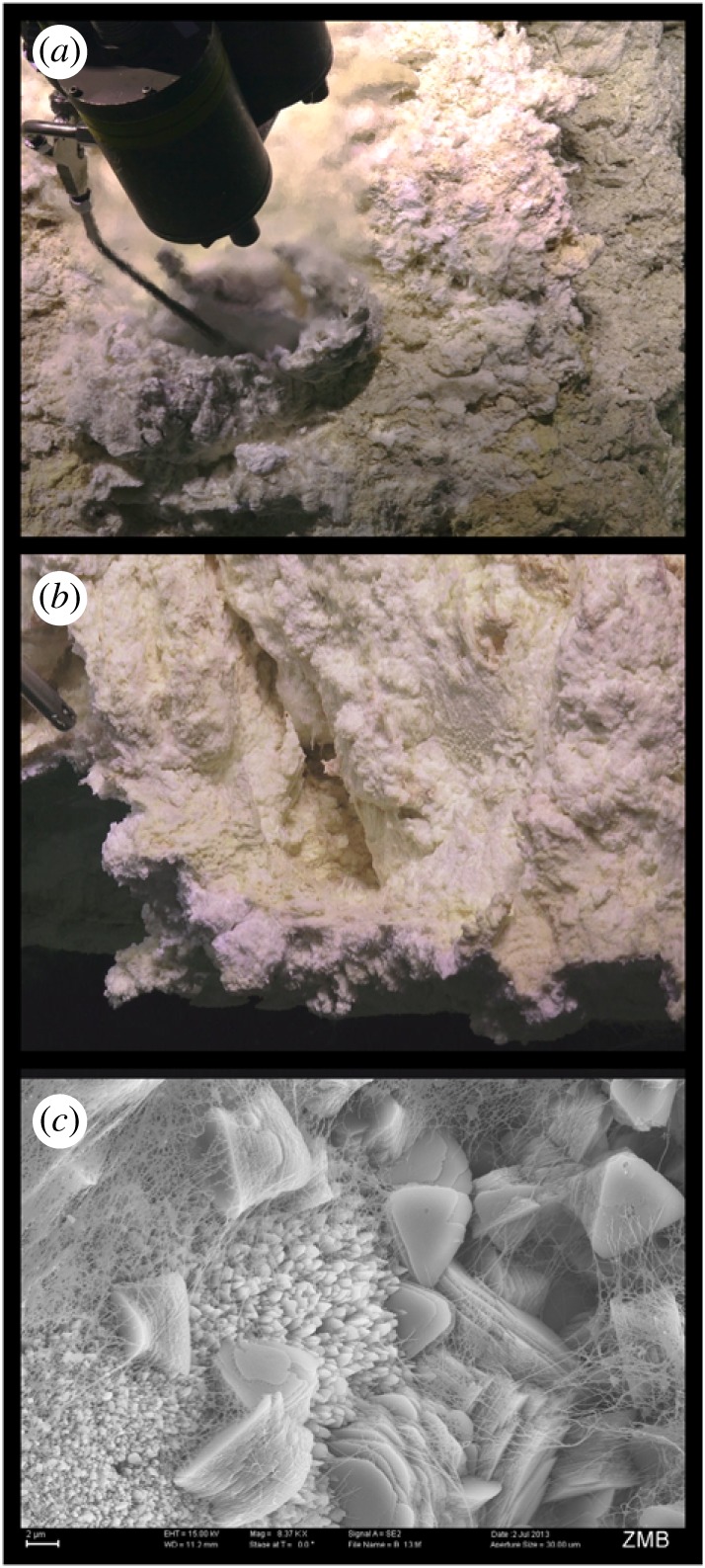
(*a*) Sampling a carbonate-brucite chimney from the Lost City that is venting fluid at approximately 95°C and pH (at 25°C) 10.8. (*b*) The exteriors of these chimneys are coated with biofilms that can be observed along the edges and in the cracks of the chimneys. Most of these biofilms inhabit conditions much more moderate than in the focused flow shown in part A. (*c*) Scanning electron microscope image of a Lost City chimney demonstrating the close interactions among carbonate (lower left and centre of image), brucite (triangular platy material) and biofilms. Image credits for (*a*) and (*b*) S. Q. Lang, U. of S.C./NSF/ROV Jason/2018 © WHOI. Image credit for (*c*) T.R.R. Bontognali, Space Exploration Inst./S.Q. Lang, U. of S.C./G.L. Früh-Green, ETH-Zürich/SNSF. (Online version in colour.)

The biological potential of serpentinization is visible in the thick, mucilaginous microbial biofilm communities that live in the chimneys of the Lost City hydrothermal field (figure 1; electronic supplemental material, video S1), perhaps the most famous and most heavily researched site of active serpentinization (30° N, 42° W; [8]). Water venting from Lost City chimneys never exceeds approximately 110°C, meaning that life may not be restricted solely by temperature in many parts of the hydrothermal system. H_2_ is highly abundant (up to 14 mM) in the Lost City chimneys, which contributes to high concentrations of methane (CH_4_) and formate [8–11]. Consequently, the Lost City biofilm communities are dominated by organisms likely to consume H_2_, CH_4_ and formate [12–14].

Thus, the thriving microbial ecosystems in the Lost City chimneys are a testament to the biological potential of serpentinization and its associated geochemical reactions. However, the Lost City chimneys are mixing zones where anoxic, high pH subsurface fluids mix with oxic seawater. The remarkable density of microbes in the chimneys (up to 10^9^ cells per gram [15]) is a product of the many physical and chemical gradients formed within the porous matrix of the chimneys when these two very different kinds of water are mixed together. Wherever and whenever these gradients can be established, the potential for biochemical and microbial activity is high [1,16].

The habitability of the rocky subsurface serpentinizing systems has been investigated with multiple drilling expeditions. The Lost City chimneys sit near the summit of the Atlantis Massif, a large seafloor mountain that has been studied during Integrated Ocean Drilling Project 304 and 305 and International Ocean Discovery Program 340T and 357. The most recent IODP expedition (exp. 357: ‘Serpentinization and Life’) collected rocks comprising the serpentinite subsurface of the Atlantis Massif with a focus on investigating links between geological, chemical and biological processes. Initial results from the first microbiological investigation of these rock cores have revealed a much lower biological content compared with the Lost City chimneys: microbial cells are nearly undetectable by microscopy in many serpentinite core samples and are typically present at densities approximately 100 cells per cm^3^ [17]. Similarly, low-cell density cells (below the detection limit of less than 10^3^ cells per cm^3^) were also found in gabbroic core samples drilled on an earlier expedition [18].

These low microbial densities suggest that the habitability of the serpentinite subsurface is limited by physical and/or geochemical conditions. However, it remains possible that the active microbial populations in serpentinite subsurface habitats are present in sparse but dense patches, most likely concentrated along pathways of the fluid flow through the basement rocks. In September 2018, we led an expedition to the Lost City to investigate the microbial activity in its serpentinite subsurface, sampling large volumes of venting hydrothermal fluids as natural windows into the zones of the serpentinite subsurface most likely to be biologically active. Fluids were also collected from one of the several borehole plugs that were installed during IODP Expedition 357 to allow direct access to the rocky subsurface. Previous expeditions to the Lost City in 2003 and 2005 focused on the rich biofilm communities of the chimneys rather than subsurface fluids, but the extensive, multidisciplinary research results from these prior expeditions also provide insights into the potential nature of the serpentinite subsurface. While analysis of the samples collected during the 2018 expedition (https://lostcity.biology.utah.edu) are underway (the public data available under BCO-DMO project no. 658604), we summarize here what is known to date about the Lost City system.

Our discussion of the many parameters relevant to understanding and predicting the habitability of serpentinite-hosted environments is structured by the requirements for habitability as summarized by Cockell *et al*. [19]:

(1) a solvent
(2) physico-chemical conditions
(3) energy
(4) major elements needed for life (CHNOPS)
(5) additional required elements, such as trace metals.

We focus here on oceanic, alkaline serpentinizing systems in particular because of the distinct role that they play in hypotheses on the emergence of life [20] and their relevance to astrobiological exploration within and beyond our solar system. While numerous serpentinite-hosted systems have been identified on the continents [21–24] and in the ocean [25,26], the most comprehensive field studies have been carried out at the Lost City hydrothermal field and its surrounding environment of the Atlantis Massif [8]. Furthermore, the geologic context of the Atlantis Massif as a relatively recent (approx. 1.5 Myr) creation of new oceanic crust, in principle, provides a more straightforward interpretation of the links between geochemical and biochemical processes. Serpentinizing systems hosted on 100–500 million-year-old continental rocks, by contrast, have complex geological histories that may complicate investigations of the origins and pathways of carbon and energy. Furthermore, oceanic systems are more representative of the ‘ocean worlds’ that arguably represent the most likely potential habitats for the extraterrestrial life [27]. Therefore, we use research results from expeditions to the Lost City and the Atlantis Massif as our bases for assessing habitability of the oceanic serpentinite subsurface.

## 2. A solvent

Liquid water is a requirement for life as we know it and may be unavailable on many extraterrestrial planetary bodies [19]. On marine systems on Earth, it is readily available throughout the hydrothermal circulation pathway, particularly when temperatures are less than 300°C, and neither vapour nor brine phases are formed. In these cases, a more significant control on the availability of water to a microbial ecosystem may be porosity and permeability [28,29].

Actively venting structures and carbonates growing from fissures in the serpentinite bedrock have high porosities of 33–57% [30], allowing ready access to liquid water. In the rocky subsurface of the Atlantis Massif, water is less readily available. Porosities of rocks recovered during drilling the Atlantis Massif on Legs 304/305 varied based on lithology, with peridotites (3.2 ± 0.3%) having somewhat higher porosities than basalts (1.9 ± 1.4%) and gabbro (2.0 ± 1.6%) [31]. On the more recent IODP Drilling Leg 357, serpentinite porosities ranged from 3.1 to 8.9%, while mafic rocks ranged from 1.8 to 2.9% [32]. These lower porosities may account in part for the cell concentrations in the subsurface of 10–10^3^ cells per cm^3^ [17], substantially lower than those in the more porous carbonate chimneys (10^9^ cells per cm^3^ [15]).

In the basaltic subsurface, cells are concentrated along fluid flow pathways [33,34], and so bulk cell count data may mask higher abundances in a subset of the subsurface. At the Lost City, fluids are thought to be channelled along faults and fractures created as a result of the detachment fault, tectonic uplift and the volume increases associated with the serpentinization reaction [8,35–37]. To accurately recreate the temperature regime of the subsurface, a relatively high permeability regime underlying the Lost City hydrothermal field (10^−14^ to 10^−15^ m^2^) is predicted to be bounded by an adjacent low permeability (less than 10^−16^ m^2^), conductive regime that creates a lateral conductive boundary layer to drive circulation [38]. These predicted subsurface regions of high seawater circulation have not yet been directly sampled, and so the degree to which they concentrate and fuel biomass remains unknown.

## 3. Physico-chemical conditions

The temperatures of endmember fluids at the Lost City are relatively moderate (40–116°C [8,39]) and do not exceed those of the currently known upper limits of life (122°C [40]). Modelling of the subsurface underlying the Lost City indicates that temperatures remain habitable to life to at least 1.2 km below the seafloor [38]. The pH of fluids when measured shipboard range from 10.1 to 10.7 [8,39,41] but, for the hottest fluids, would be approximately 8–8.5 at *in situ* temperatures and pressures [39]. Lower temperature fluids and lower pressure systems, such as continental springs, will have *in situ* pH values that are closer to the values measured at 25°C. Modelling of the approximately 40–60°C fluids at the Lost City has not yet been carried out, but given identical fluid chemistries would have higher *in situ* pH values than the hottest fluids.

**Figure 2.**
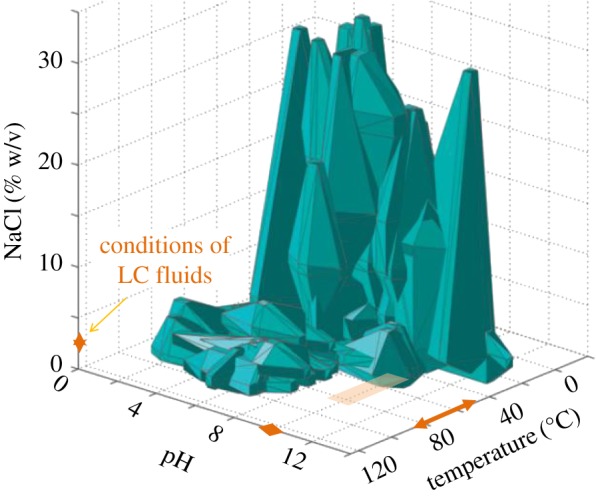
Known boundaries for life based on prokaryote cultures, adapted with permission from [42]. pH and temperature conditions for the Lost City fluids highlighted in arrows along the axes and with square shading. (Online version in colour.)

While individually neither temperature nor pH would preclude life, a review of the optimal growth conditions for cultured microorganisms has highlighted a stark gap: organisms that grow at both high pH and high temperature [42] (figure 2). The microbial adaptations required to tolerate an individual extreme such as a temperature greater than or equal to 50°C can intensify the difficulties to adapt to other extremes such as pH. ‘Polyextremophiles’ are capable of proliferating under multiple extreme conditions, and numerous examples of species adapted to higher temperatures in addition to either low pH or high salinity have been documented. No known alkalithermophiles, by contrast, are both hyperthermophilic and extremely alkaliphilic. As the optimum temperature for an organism increases, the maximum pH it can handle decreases, suggesting that the combination of high pH and high temperature is more physiologically challenging than pH or temperature alone [43]. For example, *Thermococcus alcaliphilus* is the most thermophilic of the known aklalithermophiles (optimal growth temperature of 85°C), but its optimal pH is only 9 and cannot grow above pH 10.5 [44].

Until the Lost City was discovered, no high-temperature, high-pH habitats had been studied. Indeed, the temperature and pH conditions of the Lost City span precisely the gap identified in [42]. Therefore, we posit that the absence of extremely alkaliphilic hyperthermophiles reflects a sampling and experimental bias rather than an inherent inability for microorganisms to overcome such conditions. However, due to the absence of any cultivated strains of the Lost City microbes, very little is known about their physiology [12]. Repeated attempts to cultivate the Methanosarcinales that are associated with abundant biofilms in the chimneys of both the Lost City and a similar hydrothermal system at the Prony Bay (New Caledonia) have failed to produce a pure isolate [12,45], although potential activity experiments have detected metabolism of methane at 70–80°C and pH 9–10 [12]. All isolates reported from Prony Bay are mesophilic, fermentative clostridia, and none of them can grow above pH 10 or 10.5 [46–48].

The unusual combination of warm temperature and alkaline pH is also intriguing from the perspective of the origins and early evolution of biochemical pathways. The temperature range of the Lost City chimneys (approx. 40–110°C) is ideal for many biochemical reactions, and the small compartments provided by the highly porous matrix of the carbonate chimneys can act as microscale reaction vessels [49]. Laboratory simulations have demonstrated the potential for Lost City-like conditions to favour many different chemical reactions that could have played roles during the origin and early evolution of life [50], including the reductive amination of pyruvate into the simple amino acid alanine [51] and the replication and elongation of small DNA molecules [52].

Earth's ancient ocean is expected to have been more acidic than it is today, so a proton gradient across the walls of alkaline microcompartments within the Lost City-like chimneys might have provided a ‘free’ chemiosmotic force fuelling the first biochemical pathways [53,54]. The inorganic walls of these microcompartments could have provided the scaffolding for the first cell membranes, leading to the evolution of cellular life [20]. Regardless of how the origin of life may have actually occurred on Earth, these ideas usefully highlight large gaps in knowledge of how microscale temperature and pH gradients influence biochemical and metabolic activity.

## 4. Energy

Life requires a constant flux of energy for growth and maintenance [55], supplied by steady streams of electron donors and acceptors (oxidants and reductants). In the surface ocean, organisms capitalize on the abundant energy supplied by the sun and, away from the photic zone, continue to capitalize on it by respiring photosynthetically derived organic matter with photosynthetically derived oxygen. Even in sediments and other environments where oxygen has been depleted, anaerobic organisms consume photosynthetically derived organic matter with alternative oxidants (such as sulfate and nitrate) that are still indirectly derived from photosynthesis. The presence of photosynthetic life has so fundamentally altered Earth's geochemical landscape that it is challenging to consider energy fluxes in its absence, as must have been the case on early Earth and on other planetary bodies.

Hydrothermal systems produce a steady flux of reductants that are independent of photosynthesis such as H_2_, CH_4_, H_2_S and formate. Fluids in basalt-hosted regimes lack oxidants, which restricts most chemoautotrophy to zones where reduced fluids mix with oxygenated seawater [56,57]. By contrast, the Lost City fluids contain abundant sulfate (1–4 mM [8,10]) because temperatures along the fluid pathway are not sufficiently high to fully remove it as anhydrite [8,10]. Therefore, pure endmember fluids contain millimolar concentrations of reductants (H_2_, CH_4_ and formate) and oxidants (sulfate), an energy bonanza in comparison with the majority of deep-sea habitats [58]. Indeed, we have biogeochemical and metagenomic evidence for microbial metabolism of all three of these reductants in Lost City chimneys [12,14,59]. Because this sulfate is ultimately derived from deep seawater that circulates through the hydrothermal system, it must be available throughout the fluid pathway, even in the rocky subsurface.

In short, it is unlikely that the availability of reductants and oxidants is a major limitation to habitability in present-day oceanic systems that are similar to the relatively low-temperature Lost City. Sulfate concentrations in early Earth oceans would have been substantially lower (less than 200 µM) than today [60] since concentrations rose in conjunction with increases in oxygen, but sulfate would have been present at concentrations sufficient for microbial sulfate reduction to occur [61]. Therefore, we expect that metabolic strategies such as sulfate reduction and hydrogen oxidation would not have been limited by the availability of oxidants and reductants along the fluid pathways of serpentinizing systems on the early Earth.

The constant flux of reductants from serpentinizing systems is another reason why they are appealing for many origin of life scenarios [54]. Many lines of biochemical, phylogenetic and genomic evidence point to hydrogen metabolism as ancient [62] and perhaps a key feature of the last universal common ancestor [63]. If so, it seems that serpentinizing systems, which produce millimolar quantities of H_2_ [8] and host organisms with highly abundant and diverse hydrogenase enzymes [59], are likely to have played a role in the early evolution of biochemical pathways.

## 5. Major elements for life

### (a) Bioaccessible carbon

**Figure 3.**
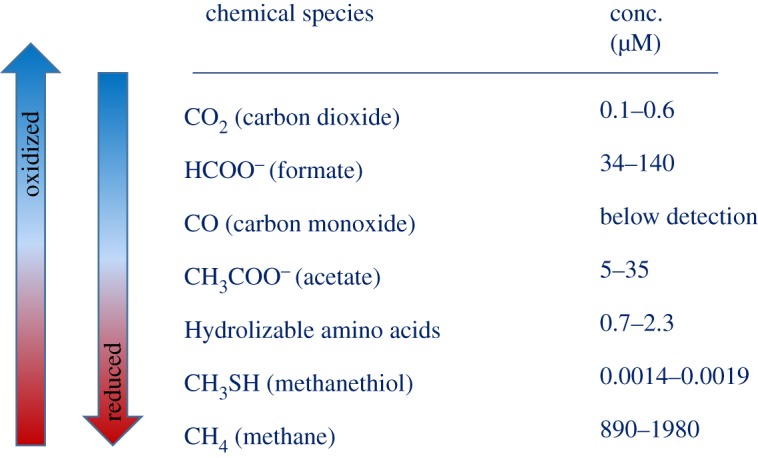
Concentrations of major known carbon species in endmember Lost City fluids. Data from [9–11,68,69]. (Online version in colour.)

An important limitation on microbial activity in the serpentinite subsurface may be the availability of $\Sigma \text{C}{\text{O}}_{2}(\Sigma \text{C}{\text{O}}_{2}=\text{C}{{\text{O}}_{2}}_{\left(aq\right)}+{\text{H}}_{2}\text{C}{\text{O}}_{3}+\text{HC}{{\text{O}}_{3}}^{-}+\text{C}{{\text{O}}_{3}}^{-2}$). Marine low-temperature alkaline serpentinization environments such as Lost City and Prony Bay and analogous land-based environments in Oman, California and Italy are characterized by extremely low concentrations of inorganic carbon due to the rapid precipitation of calcium carbonate at pHs above approximately 9 and/or its reduction to hydrocarbons [8,9,21,22,25,35,64,65]. The lack of ΣCO_2_ in these alkaline fluids and their association with carbonate formation is so notable that such systems have been proposed as a means to sequester atmospheric CO_2_ [66]. Concentrations of ΣCO_2_ at the Lost City in vents across the field are 0.1–0.6 µM with the exception of one location (Marker 3) where they are an order of magnitude higher (10–26 µM), and independent lines of evidence indicate that seawater is entrained into fluids in the near-subsurface [9,67] (figure 3).

The next most oxidized forms of carbon in endmember fluids are the organic acids, formate and acetate [10]. Formate, a single carbon organic acid, is the second most abundant carbon-containing compound in Lost City fluids. In the presence of H_2_, formate can readily exchange with $\Sigma \text{C}{\text{O}}_{2}\;(\text{e}\text{.g}\text{. HC}{{\text{O}}_{3}}^{-}+{\text{H}}_{2}=\text{CHO}{\text{O}}^{-}+{\text{H}}_{2}\text{O}$). Thermodynamic calculations and laboratory studies indicate that this conversion is thermodynamically favourable and kinetically rapid abiotically at temperatures greater than 175°C [70–73]. At lower temperatures, microorganisms carry out the same reaction rapidly and reversibly [74,75]. A recent investigation into the isotopic (^13^C, ^14^C) signatures of formate in Lost City fluids indicates that most of the formate in endmember fluids is formed abiotically in the subsurface of the field [14]. Several lines of evidence also point to microorganisms using the formate dehydrogenase enzyme to rapidly equilibrate between CO_2_ and formate [14].

Acetate is also present in Lost City fluids, but unlike formate, the highest concentrations are in fluids that are most impacted by microbial sulfate reduction [10], and isotopic signatures are consistent with this compound being derived from the biological activity [14].

**Figure 4.**
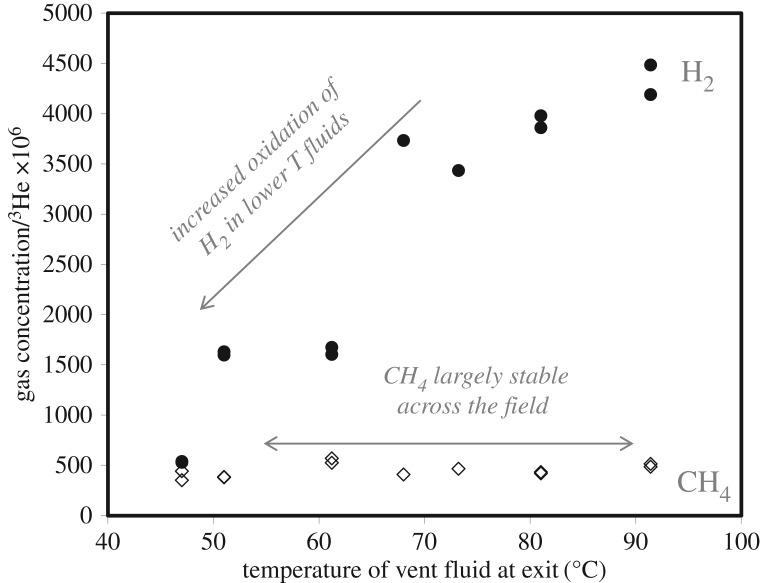
Lost City H_2_ and CH_4_ concentrations normalized to the concentration of ^3^He. Data from [9,11].

Methane (CH_4_) is by far the most abundant carbon-containing compound in Lost City fluids (0.9–2.0 mM) [8,9]. The lack of radiocarbon in Lost City CH_4_ has been used to argue that it is produced abiotically in the subsurface from ^14^C-free mantle carbon. The concentration of CH_4_ in endmember fluids varies twofold, reflecting differences in the amount of mantle input (figure 4). The addition of mantle volatiles is evident from elevated ^3^He concentrations in the fluids, and this species covaries positively with CH_4_ (*r*^2^ = 0.83, *p* < 0.01 excluding one outlier; data from [9]). This relationship may result from increasing carbon availability with the increasing mantle input; the contribution of mantle volatiles will include mantle CO_2_, which could then be converted to CH_4_. The conversion may be carried out within fluid inclusions, with the volatiles getting extracted into hydrothermal fluids during later circulation [76,77]. The strong relationship between ^3^He and CH_4_ has been used to argue that carbon limitation controls the *δ*^13^C values of the CH_4_, resulting in values similar to those of the inferred starting material [9]. The same line of argument has been used to account for the *δ*^13^C values of the short-chain hydrocarbons (−13.1 to −16.9‰; [9]) which are enriched in ^13^C compared with most oceanic organic matter (approx. −22‰).

**Figure 5.**
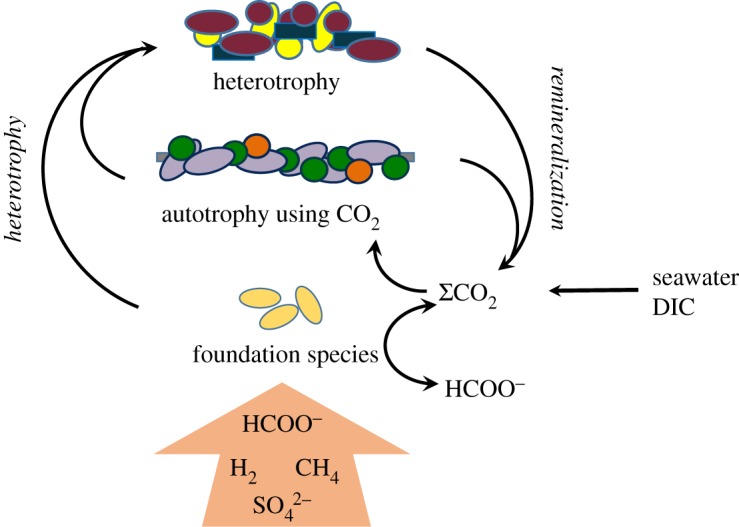
Schematic of potential carbon and microbe relationship in the Lost City chimneys, adapted from [14]. Since ΣCO_2_ is extremely scarce in the fluids of alkaline serpentinization systems, the growth of chemolithoautotrophic organisms may require the presence of a heterotrophic foundation species that supplies bioaccessible inorganic carbon to the community. We hypothesize that at the Lost City sulfate reducers convert abiotic formate into CO_2_ that is then used by species such as the Lost City Methanosarcinales to carry out hydrogenotrophic methanogenesis. (Online version in colour.)

The limited availability of ΣCO_2_ may limit the growth of chemolithoautotrophs in alkaline serpentinization systems. In addition to the low concentrations discussed above, precipitation of inorganic carbon as CaCO_3_ may have an additional impact on its bioaccessibility, according to a recent study of methanogens grown in alkaline conditions [78]. Therefore, concentrations alone may not fully reflect the availability of ΣCO_2_ to the microorganisms. Biomass and biomarkers in the carbonate chimneys of the Lost City have ^14^C signatures that indicate the extensive incorporation of mantle-derived carbon [41]. Given the evidence of the rapid microbial conversion of formate and ΣCO_2_, a likely scenario is that chemolithoautotrophs rely on the liberation of inorganic carbon from mantle-derived carbon compounds to carry out their metabolism (figure 5, [14]).

In the rocky subsurface of the Atlantis Massif, organic carbon content is generally very low, and calcium carbonate precipitates are extensive [17,32,79]. In drill cores from the Iberian Margin, higher concentrations of organic carbon are associated with locations that have been strongly altered by fluid flow [80] and have evidence of microbially derived lipid biomarkers [81]. In a drill core sample from the Atlantis Massif, the amino acid tryptophan was detected and proposed to be abiotically derived [82]. It, along with additional unidentified organic compounds [82,83], was closely associated with saponites, which form at temperatures below 150°C during aqueous alteration of serpentinized ultramafic rocks. Biologically derived compounds have also been detected in Atlantis Massif rocks and proposed to have been deposited from seawater organic matter during circulation [79]. Therefore, late-stage fluid circulation may have an important role in altering the organic content of the rocky subsurface. Given the heterogeneous distribution of these admittedly tiny amounts of organic compounds [83], it is as yet unclear how important they would be for sustaining subseafloor ecosystems.

### (b) Potential implications of carbon availability on ecosystem structure

If the limitation to biological activity in an ecosystem is the combination of temperature and pH, then every individual in the habitat must make the necessary physiological adjustments for the survival. The same is not true if the limitation is carbon. In that case, a single foundation species could be sufficient to transform a carbon source that other microorganisms cannot metabolize into biologically available carbon that supports the rest of the ecosystem. For example, a microorganism that uses abiotic formate would release ΣCO_2_ that could then be locally consumed by a hydrogen-oxidizing chemolithoautotroph incapable of using formate (figure 5). The biomass it synthesizes could also serve a heterotrophic community that would otherwise have no organic matter to decompose. In essence, this foundation species is the primary producer of the ecosystem, although in a somewhat unusual context since its carbon source is already in the organic form (i.e. formate or CH_4_). In such a scenario, the success of the single organotrophic foundation species could have broad biogeochemical consequences.

Although our current research on the Lost City chimneys has pointed to formate as the key carbon molecule, other studies and other locations may identify other molecules as more important in particular circumstances. Nevertheless, it seems that the fundamental chemistry of serpentinizing environments will lead to a scarcity of ΣCO_2_ that causes heterotrophic, not autotrophic, organisms to be the foundations of these ecosystems. Indeed, the primary colonizers of newly formed Lost City-like chimneys in Prony Bay (New Caledonia) are likely to be heterotrophic bacteria [84]. Clostridia and their relatives appear to be typical of subsurface systems [85], but some species (e.g. *Desulfotomaculum*) are also capable of using H_2_ as an electron donor and sulfur compounds as electron acceptors [86]. Since these organisms, and probably others as well, can use organic carbon compounds as their carbon source but might supplement their energy budget with electrons from H_2_, it will be important for the future research in serpentinizing systems to separate how organisms obtain their energy (lithotrophic versus organotrophic) from how they obtain their carbon (autotrophic versus heterotrophic).

Therefore, the biological potential of the serpentinite subsurface may be determined by its ability to metabolize simple organic molecules such as formate or CH_4_; without this capability, biological activity in the serpentinite subsurface would be limited to those regions with extensive exposure to circulating seawater, significantly limiting the habitable volume of the subsurface of Earth. However, it seems reasonable to expect that life would have found a way to exploit the essentially free source of organic carbon generated in serpentinites, especially if an oxidant such as sulfate is readily available.

### (c) Nitrogen

Very few measurements have been conducted on the abundance of dissolved nitrogen in endmember serpentinizing hydrothermal fluids. Concentrations of both nitrate and ammonia are less than 6 µM in endmember fluids at the Lost City [68], while seawater nitrate concentrations in the Atlantic Ocean at the depths of the field are approximately 10–25 µM [87,88]. Similar to basalt-hosted systems, the elevated temperatures and reducing conditions of the serpentinite subsurface, possibly in combination with subsurface microbial activity, can lead to a removal of nitrate and/or the production of ammonia [89].

The biological communities in the carbonate chimneys do not appear to be nitrogen limited, however. Total hydrolyzable amino acid concentrations of endmember fluids are higher than found in the background seawater (0.7–2.3 µM versus 0.3 µM [68]). Most carbonate-brucite chimneys have organic C/N ratios of 4.1–6.7 [68], a value consistent with that of marine bacteria (4–6 [90–92] and methanogens (approx. 7 [93]).

Microbial communities within the chimneys may be able to satisfy their nitrogen demands with seawater nitrate due to the extensive mixing within the chimneys. Biological nitrogen fixation could also contribute to sources of fixed nitrogen. The ^15^N of the organic matter in the chimneys ranges from 0.1 to 5.6‰, potentially reflecting nitrogen fixation, which results in minimal fractionation of the starting N_2_ [68]. In deep seawater, N_2_ has a value of approximately −2 to 0‰ [94,95], while the isotopic signature of N_2_ in the mantle has been estimated as −5 ± 2‰ from oceanic basalts [96,97]. The isotopic signature of N_2_ within serpentinite-hosted systems has not been determined to our knowledge. Nitrogen fixation genes, including a sequence identical to a hyperthermophilic nitrogen-fixing methanogen, have been identified in the metegenomic data from the Lost City chimneys [12,98].

The N content of rocks in the subsurface is likely very low. Rock core samples collected during IODP Expedition 357 had less than 50 ppm N, including basalts, serpentinites and gabbros (S.Q. Lang, unpublished data, 2019). This result is consistent with the recent observation that the N in a serpentinite wedge (5–45 ppm) was likely sourced from fluids that passed through subducted marine sediments [99] as well as observations that serpentinite soils generally lack N [100]. Genes coding for nitrogen fixation, ammonium oxidation and nitrate reduction were observed in sequences from gabbroic and basaltic samples recovered during a previous drilling expedition to the Atlantis Massif [18], suggesting that the subsurface community may be capable of synthesizing and accessing fixed nitrogen.

### (d) Phosphorous

While phosphorous is an essential component of all living organisms, at this time, we are not aware of the published data on its concentrations in fluids, chimneys or rocks from the Atlantis Massif. In basalt-hosted systems, phosphate is removed as a result of sorption onto iron-rich minerals and due to reactions among seawater, basalt and sediments [101,102]. The removal is more extensive at higher temperatures and potentially incomplete at temperatures less than approximately 80°C [101]. Endmember fluids may be phosphate limited at the Lost City, depending on iron abundances and the temperatures reached during fluid circulation. In the chimneys at Lost City, archaeal and bacterial lipid biomarkers that typically have a phosphorous-containing head group instead have a glycosyl head group that does not require phosphorous, possibly as an adaptation to low abundances of this nutrient [103].

Similar to nitrate availability, access to phosphate may be dependent on near-surface mixing with oxygenated seawater. Once phosphate enters the system, it is likely to be trapped and concentrated in porous mineral deposits, such as the Lost City chimneys. The concentration of phosphate-bearing minerals in rocky habitats could have been a key aspect of early molecular evolution [85,104,105].

### (e) Sulfur

The fluids from the Lost City are unusual in that a substantial concentration of sulfate (1.0–3.7 mM; [8,41]) is present in the highly reducing endmember fluids. In most hydrothermal fluids, the sulfate carried with recharge water is removed as anhydrite during circulation. It may be that the maximum temperature that the Lost City fluids reach in the subsurface is not sufficient for the complete removal of sulfate as anhydrite.

Bisulfide (HS^−^) is also present in the fluids, with concentrations ranging from 0.2 to 2.9 mM [8,41]. Across the field, concentrations of sulfide increase in conjunction with decreases in sulfate and hydrogen, clearly demonstrating the consequences of sulfate reduction in the subsurface. At this time, it is not clear why some fluids have been extensively altered by sulfate reduction than others, although temperature may play a role. The fluids with the lowest sulfide concentrations (0.2–0.3 mM; Markers 6, 3, BH) have higher temperatures than the fluids with higher sulfide (0.9–2.9 mM; Markers C, 7, 8, 2, H) (68–91°C versus 47–73°C) [41]. Hydrogen concentrations are also higher in warmer fluids (figure 4; data from [11]), as are sulfate concentrations. The trend with temperature may also indicate a temperature limitation of biological sulfate reduction in the subsurface; at lower temperatures and/or longer residence times of fluid circulation, greater subsurface microbial activity could result in the conversion of hydrogen and sulfate into sulfide.

Sulfur is also abundant in the serpentinite subsurface (28–7221 ppm), in the form of both sulfide minerals and sulfate [106]. Much of this sulfur is ultimately derived from seawater sulfate that is incorporated into the rock during hydrothermal circulation [106]. By contrast, depleted mantle peridotite contains approximately 60–120 ppm of sulfur [107].

## 6. Additional required elements

### (a) Trace metals and other elements

Trace metals such as iron, nickel and molybdenum are bound to key enzymes in metabolic pathways and are thus essential for the microbial growth [108]. Concentrations of trace metals in the alkaline fluids and carbonate-brucite chimneys of the Lost City rarely exceed detection limits, and their scarcity may pose a major challenge to growth [8,30,39]. Nevertheless, methanogens are abundant in the Lost City chimney biofilms [15] despite having an enzymatic pathway with a high requirement for trace metals [109,110]. Unlike many of the other requirements for life that would seem to favour the chimney environment, trace metal abundances are far higher in the rocky subsurface.

Actively venting structures have low concentrations of trace metals compared with those that are no longer exposed to hydrothermal fluid flow and bathed in seawater at more moderate temperatures [8,30]. For example, Mn concentrations increase from an average of 4.1 ppm in active chimneys to 42.1 ppm in inactive structures, likely due to the formation of Mn-oxides and Mn uptake during calcite precipitation [30]. Similarly, other trace metals such as Co, Cr, V, Ti and Ni are below detection (0.82, 23.37, 3.37, 73.32 and 16.11 ppm, respectively) in active structures but reach concentrations of greater than 40 ppm due to incorporation into inactive chimneys [30].

In all cases, iron concentrations in the chimneys are below the detection limit of 50 ppm [30]. A maximum of approximately 3 µM of iron is present in the fluids although these data should be treated with some caution due to difficulties in sampling [39]. Endmember concentrations of several trace alkali elements are higher in endmember fluids than in seawater, including Li (approx. 40 versus 24 µM), Rb (2.8 versus 1.4 µM) and Cs (17 versus 2.3) [39].

In contrast to the fluids and the carbonate-brucite chimneys, the rocky subsurface of the Atlantis Massif is rich in trace metals. Rocks recovered from drilling contain elevated concentrations of Co (33–622 ppm), Cr (100–29 698 ppm), V (18.6–340 ppm), Ni (94–14 590 ppm) and Fe_2_O_3_ (3.4–32.1 weight %) [32]. The abundance of trace metals at levels that are 100 to 1000 times higher than in the carbonate chimneys makes it unlikely that their availability would limit life in the subsurface.

Therefore, trace metals may be limiting in the otherwise rich microbial ecosystems of the Lost City chimneys, but the obvious abundance of life in these metal-poor habitats indicates that the delivery of metals from the subsurface to surface, or from mixing with seawater, must be sufficient. However, it is unknown whether the chimney biofilms accumulate and store metals as they flow through the chimneys, and whether these metal stores are extracellular and therefore serve as public goods within the community. An alternative possibility is that the individual organisms have simply adapted to a low-metal lifestyle. The strategies that enable biofilm communities to thrive in the low-metal conditions of the Lost City chimneys remain unexplored.

## 7. Conclusion

The evolution and maintenance of a robust biological ecosystem requires continuous habitability over geological time scales [19]. The absence of certain elemental requirements (e.g. a solvent, energy, appropriate physico-chemical conditions, major and minor elements for life) will preclude life from being able to maintain its complex state. But simply meeting the criteria may also be insufficient to achieve habitability. Rather, amounts of resources also matter for each parameter individually and in combination [55]. In serpentinizing systems, life may not be limited by a single limiting factor, but instead by combinations of factors that cannot be overcome simultaneously.

Marine serpentinizing systems are unusual in that life is not limited by energy availability, unlike the majority of deep subseafloor environments that are constrained by oxidant availability (e.g. continental shelf sediments) or reductant availability (e.g. abyssal plain sediments). In some ways, they are analogous to highly productive surface ocean ecosystems, where primary production is limited by the availability of nutrients and trace metals, not energy.

What, then, is the limit to habitability in serpentinizing systems? As discussed above, the answer may depend on which component of the system is considered. Throughout the fluid pathway, life will be challenged by low-to-absent bioavailable ΣCO_2_ and the combination of elevated temperatures and pHs. In the rocky subsurface, nitrogen availability may present an additional challenge, while the carbonate chimneys may lack trace metals. The role of seawater mixing for the delivery of ΣCO_2,_ trace metals and nitrogen in the chimneys and in the subsurface remains unclear. In addition, the combination of high temperature and high pH throughout the serpentinizing system is likely to amplify the physiological stress associated with coping with each of these potential limitations.

Extending this perspective of the habitability of marine serpentinizing systems to early Earth or other planetary bodies is speculative. Sites of serpentinization beyond Earth may lack an ocean (e.g. Mars [111]), or vigorous hydrothermal circulation, or other key requirements in the circulating water. Sulfate concentrations in extraterrestrial oceans are likely to be substantially lower, shifting the energy landscape. Even if an ocean world is full of oxidants and reductants, another fundamental question is whether serpentinization can sustain a hydrothermal circulation system without the energy of magmatism and tectonics. At the Lost City, heat released from the cooling lithosphere appears necessary to drive fluid circulation [38,112].

If habitability relies on the circulation of fluids through the subsurface to continually deliver ΣCO_2_, phosphorous, nitrogen and trace metals, a key question becomes the nature of that circulation. On what time scale can circulation be maintained, and is the circulation regional or global [6,27]? Is the source of oxidants strong and sustainable enough to maintain an ecosystem [113]? Many of these questions are currently being addressed with on-going observations of the potential global oceans on Europa and Enceladus [4–6].

More broadly, major questions remain in assessing the habitability of serpentinizing systems. To what extent does a ‘combination of stressors' pose a problem to life? Can these stressors be overcome with abundant energy? During circulation, what is the timing of the production of reductants (H_2_ and CH_4_), the elevation of pH, loss of ΣCO_2_ and rise in temperatures? Is exposing fresh rock and mineral surfaces to circulating fluids necessary for maintaining a steady output of reductants? Is the mixing of serpentinizing fluids with seawater necessary to produce a gradient of temperature, pH and other parameters that enables multiple combinations of habitable conditions? For how long can an isolated subsurface system maintain habitability without regional or global circulation of water?

Filling these knowledge gaps is important not only for a better understanding of the evolution and distribution of life on Earth but also for the search and detection of life elsewhere. Potential extraterrestrial habitats are best evaluated on the basis of their ability to support a robust ecosystem, not a lone organism, over geological time scales [114]. Future work in this area requires a better understanding of how multiple extreme limitations of life interact to restrict, or perhaps even promote, habitability in unusual and complex environments.

## Supplementary Material

Video S1. A small chimlet at the Lost City hydrothermal field

## Data accessibility

This article does not contain any additional data.

## References

[1] McCollom TM, Seewald JS 2007 Abiotic synthesis of organic compounds in deep-sea hydrothermal environments. Chem. Rev. 107, 382–401. (10.1021/cr0503660) 17253758

[2] Martin W, Baross J, Kelley D, Russell MJ 2007 Hydrothermal vents and the origin of life. Nat. Rev. Microbiol. 6, 805–814. (10.1038/nrmicro1991) 18820700

[3] Sleep NH, Meibom A, Fridriksson T, Coleman RG, Bird DK 2004 H-2-rich fluids from serpentinization: geochemical and biotic implications. Proc. Natl Acad. Sci. USA 101, 12 818–12 823. (10.1073/pnas.0405289101) PMC516479 15326313

[4] Glein CR, Baross JA, Waite JH 2015 The pH of Enceladus' ocean. Geochim. Cosmochim. Acta 162, 202–219. (10.1016/j.gca.2015.04.017)

[5] Waite JH et al 2017 Cassini finds molecular hydrogen in the Enceladus plume: evidence for hydrothermal processes. Science 356, 155–159. (10.1126/science.aai8703) 28408597

[6] Vance SD, Hand KP, Pappalardo RT 2016 Geophysical controls of chemical disequilibria in Europa. Geophys. Res. Lett. 43, 4871–4879. (10.1002/2016GL068547)

[7] Stueken EE, Anderson RE, Bowman JS, Brazelton WJ, Colangelo-Lillis J, Goldman AD, Som SM, Baross JA 2013 Did life originate from a global chemical reactor? Geobiology 11, 101–126. (10.1111/gbi.12025) 23331348

[8] Kelley DS et al 2005 A serpentinite-hosted ecosystem: the Lost City hydrothermal field. Science 307, 1428–1434. (10.1126/science.1102556) 15746419

[9] Proskurowski G, Lilley MD, Seewald JS, Früh-Green GL, Olson EJ, Lupton JE, Sylva SP, Kelley DS 2008 Abiogenic hydrocarbon production at Lost City hydrothermal field. Science 319, 604–607. (10.1126/science.1151194) 18239121

[10] Lang SQ, Butterfield DA, Schulte M, Kelley DS, Lilley MD 2010 Elevated concentrations of formate, acetate and dissolved organic carbon found at the Lost City hydrothermal field. Geochim. Cosmochim. Acta 74, 941–952. (10.1016/j.gca.2009.10.045)

[11] Proskurowski G, Lilley MD, Kelley DS, Olson EJ 2006 Low temperature volatile production at the Lost City hydrothermal field, evidence from a hydrogen stable isotope geothermometer. Chem. Geol. 229, 331–343. (10.1016/j.chemgeo.2005.11.005)

[12] Brazelton WJ, Mehta MP, Kelley DS, Handelsman J 2011 Physiological differentiation within a single-species biofilm fueled by serpentinization. Mbio 2, 1–9. (10.1128/mBio.00127-11) PMC3143844 21791580

[13] Brazelton W, Schrenk M, Kelley D, Baross JA 2006 Methane- and sulfur-metabolizing microbial communities dominate the Lost City hydrothermal field ecosystem. Appl. Environ. Microbiol. 72, 6257–6270. (10.1128/AEM.00574-06) 16957253 PMC1563643

[14] Lang SQ, Fruh-Green GL, Bernasconi SM, Brazelton WJ, Schrenk MO, McGonigle JM 2018 Deeply-sourced formate fuels sulfate reducers but not methanogens at Lost City hydrothermal field. Sci. Rep. 8, 755 (10.1038/s41598-017-19002-5) 29335466 PMC5768773

[15] Schrenk MO, Kelley DS, Bolton SA, Baross JA 2004 Low archaeal diversity linked to subseafloor geochemical processes at the Lost City hydrothermal field, mid-Atlantic ridge. Environ. Microbiol. 6, 1086–1095. (10.1111/j.1462-2920.2004.00650.x) 15344934

[16] Summit M, Baross JA 2001 A novel microbial habitat in the mid-ocean ridge subseafloor. Proc. Natl Acad. Sci. USA 98, 2158–2163. (10.1073/pnas.051516098) 11226209 PMC30109

[17] Fruh-Green GL et al 2018 Magmatism, serpentinization and life: insights through drilling the Atlantis Massif (IODP Expedition 357). Lithos 323, 137–155. (10.1016/j.lithos.2018.09.012)

[18] Mason OU, Nakagawa T, Rosner M, Van Nostrand JD, Zhou J, Maruyama A, Fisk MR, Giovannoni SJ 2010 First investigation of the microbiology of the deepest layer of ocean crust. PLoS ONE 5, e15399 (10.1371/journal.pone.0015399) 21079766 PMC2974637

[19] Cockell CS et al 2016 Habitability: a review. Astrobiology 16, 89–117. (10.1089/ast.2015.1295) 26741054

[20] Martin W, Russell MJ 2007 On the origin of biochemistry at an alkaline hydrothermal vent. Phil. Trans. R. Soc. B 362, 1887–1925. (10.1098/rstb.2006.1881) 17255002 PMC2442388

[21] Barnes I, O'Neil JR, Trescases JJ 1978 Present day serpentinization in New Caledonia, Oman and Yugoslavia. Geochim. Cosmochim. Acta 42, 144–145. (10.1016/0016-7037(78)90225-9)

[22] Neal C, Stanger G 1983 Hydrogen generation from mantle source rocks in Oman. Earth Planet. Sci. Lett. 66, 315–320. (10.1016/0012-821X(83)90144-9)

[23] Szponar N, Brazelton WJ, Schrenk MO, Bower DM, Steele A, Morrill PL 2013 Geochemistry of a continental site of serpentinization, the Tablelands Ophiolite, Gros Morne National Park: a Mars analogue. Icarus 224, 286–296. (10.1016/j.icarus.2012.07.004)

[24] Bruni J, Canepa M, Chiodini G, Cioni R, Cipolli F, Longinelli A, Marini L, Ottonello G, Vetuschi Zuccolini M 2002 Irreversible water-rock mass transfer accompanying the generation of the neutral, Mg–HCO_3_ and high-pH, Ca–OH spring waters of the Genova province, Italy. Appl. Geochem. 17, 455–474. (10.1016/S0883-2927(01)00113-5)

[25] Monnin C et al 2014 Fluid chemistry of the low temperature hyperalkaline hydrothermal system of Prony Bay (New Caledonia). Biogeosciences 11, 5687–5706. (10.5194/bg-11-5687-2014)

[26] Ohara Y et al 2012 A serpentinite-hosted ecosystem in the Southern Mariana Forearc. Proc. Natl Acad. Sci. USA 109, 2831–2835. (10.1073/pnas.1112005109) 22323611 PMC3286937

[27] Vance S, Harnmeijer J, Kimura J, Hussmann H, Demartin B, Brown JM 2007 Hydrothermal systems in small ocean planets. Astrobiology 7, 987–1005. (10.1089/ast.2007.0075) 18163874

[28] Tanikawa W, Tadai O, Morono Y, Hinrichs KU, Inagaki F 2018 Geophysical constraints on microbial biomass in subseafloor sediments and coal seams down to 2.5 km off Shimokita Peninsula, Japan. Progress Earth Planetary Sci. 5, 58 (10.1186/s40645-018-0217-2)

[29] Fredrickson JK, Garland TR, Hicks RJ, Thomas JM, Li SW, Mcfadden KM 1989 Lithotrophic and heterotrophic bacteria in deep subsurface sediments and their relation to sediment properties. Geomicrobiol. J. 7, 53–66. (10.1080/01490458909377849)

[30] Ludwig KA, Kelley DS, Butterfield DA, Nelson BK, Früh-Green G 2006 Formation and evolution of carbonate chimneys at the Lost City hydrothermal field. Geochim. Cosmochim. Acta 70, 3625–3645. (10.1016/j.gca.2006.04.016)

[31] Scientists E, Site U1309. 2006 In Hrsg. Proc. Integrated Ocean Drilling Program (eds Blackman DK, Ildefonse B, John BE et al.). College Station, TX: Integrated Ocean Drilling Program Management International, Inc.

[32] Fruh-Green GL et al. Eastern sites. 2017 In Hrsg. Proc. Int. Ocean Discovery Program (eds Fruh-Green GL, Orcutt BN, Green SL et al.).

[33] Labonte JM, Lever MA, Edwards KJ, Orcutt BN 2017 Influence of igneous basement on deep sediment microbial diversity on the eastern Juan de Fuca Ridge Flank. Front. Microbiol. 8, 1434 (10.3389/fmicb.2017.01434) 28824568 PMC5539551

[34] Smith A et al 2011 *In situ* enrichment of ocean crust microbes on igneous minerals and glasses using an osmotic flow-through device. Geochem. Geophys. Geosyst. 12, 1–19. (10.1029/2010gc003424)

[35] Fruh-Green GL, Connolly JAD, Plas A, Kelley DS, Grobéty B 2004 Serpentinization of oceanic peridotites: implications for geochemical cycles and biological activity. In Subseafloor biosphere at mid-ocean ranges, vol. 144 (eds WSD Wilcock, EF Delong, DS Kelley, JA Baross, SC Cary), pp. 119–136. (10.1029/144GM08)

[36] Karson JA, Fruh-Green GL, Kelley DS, Williams EA, Yoerger DR, Jakuba M 2006 Detachment shear zone of the Atlantis Massif core complex, Mid-Atlantic Ridge, 30° N. Geochem. Geophys. Geosyst. 7, 1–29. (10.1029/2005gc001109)

[37] Boschi C, Fruh-Green GL, Delacour A, Karson JA, Kelley DS 2006 Mass transfer and fluid flow during detachment faulting and development of an oceanic core complex, Atlantis Massif (MAR 30°N). Geochem. Geophys. Geosyst. 7, 1–39. (10.1029/2005GC001074)

[38] Titarenko SS, McCaig AM 2016 Modelling the Lost City hydrothermal field: influence of topography and permeability structure. Geofluids 16, 314–328. (10.1111/gfl.12151)

[39] Seyfried WE, Pester NJ, Tutolo BM, Ding K 2015 The Lost City hydrothermal system: constraints imposed by vent fluid chemistry and reaction path models on subseafloor heat and mass transfer processes. Geochim. Cosmochim. Acta 163, 59–79. (10.1016/j.gca.2015.04.040)

[40] Takai K et al 2008 Cell proliferation at 122°C and isotopically heavy CH_4_ production by a hyperthermophilic methanogen under high-pressure cultivation. Proc. Natl Acad. Sci. USA 105, 10 949–10 954. (10.1073/pnas.0712334105) PMC2490668 18664583

[41] Lang SQ, Fruh-Green GL, Bernasconi SM, Lilley MD, Proskurowski G, Méhay S, Butterfield DA 2012 Microbial utilization of abiogenic carbon and hydrogen in a serpentinite-hosted system. Geochim. Cosmochim. Acta 92, 82–99. (10.1016/j.gca.2012.06.006)

[42] Harrison JP, Gheeraert N, Tsigelnitskiy D, Cockell CS 2013 The limits for life under multiple extremes. Trends Microbiol. 21, 204–212. (10.1016/j.tim.2013.01.006) 23453124

[43] Wiegel J, Kevbrin VV 2004 Alkalithermophiles. Biochem. Soc. Trans. 32, 193–198. (10.1042/bst0320193) 15046570

[44] Keller M, Braun FJ, Dirmeier R, Hafenbradl D, Burggraf S, Rachel R, Stetter KO 1995 *Thermococcus alcaliphilus* sp. nov., a new hyperthermophilic archaeum growing on polysulfide at alkaline pH. Arch. Microbiol. 164, 390–395. (10.1007/BF02529736) 8588740

[45] Postec A et al 2015 Microbial diversity in a submarine carbonate edifice from the serpentinizing hydrothermal system of the Prony Bay (New Caledonia) over a 6-year period. Front. Microbiol. 6, 857 (10.3389/fmicb.2015.00857) 26379636 PMC4551099

[46] Ben Aissa F, Postec A, Erauso G, Payri C, Pelletier B, Hamdi M, Fardeau M-L, Ollivier B 2015 Characterization of *Alkaliphilus hydrothermalis* sp. nov., a novel alkaliphilic anaerobic bacterium, isolated from a carbonaceous chimney of the Prony hydrothermal field, New Caledonia. Extremophiles 19, 183–188. (10.1007/s00792-014-0697-y) 25319677

[47] Mei N, Postec A, Erauso G, Joseph M, Pelletier B, Payri C, Ollivier B, Quéméneur M 2016 *Serpentinicella alkaliphila* gen. nov., sp. nov., a novel alkaliphilic anaerobic bacterium isolated from the serpentinite-hosted Prony hydrothermal field, New Caledonia. Int. J. Syst. Evol. Microbiol. 66, 4464–4470. (10.1099/ijsem.0.001375) 27499124

[48] Bes M et al 2015 *Acetoanaerobium pronyense* sp. nov., an anaerobic alkaliphilic bacterium isolated from a carbonate chimney of the Prony hydrothermal field (New Caledonia). Int. J. Syst. Evol. Microbiol. 65, 2574–2580. (10.1099/ijs.0.000307) 25948619

[49] Baaske P, Weinert FM, Duhr S, Lemke KH, Russell MJ, Braun D 2007 Extreme accumulation of nucleotides in simulated hydrothermal pore systems. Proc. Natl Acad. Sci. USA 104, 9346–9351. (10.1073/pnas.0609592104) 17494767 PMC1890497

[50] Barge LM, White LM 2017 Experimentally testing hydrothermal vent origin of life on Enceladus and other icy/ocean worlds. Astrobiology 17, 820–833. (10.1089/ast.2016.1633) 28836818

[51] Barge LM, Flores E, Baum MM, Vandervelde DG, Russell MJ 2019 Redox and pH gradients drive amino acid synthesis in iron oxyhydroxide mineral systems. Proc. Natl Acad. Sci. USA 116, 4828–4833. (10.1073/pnas.1812098116) 30804197 PMC6421445

[52] Kreysing M, Keil L, Lanzmich S, Braun D 2015 Heat flux across an open pore enables the continuous replication and selection of oligonucleotides towards increasing length. Nat. Chem. 7, 203–208. (10.1038/nchem.2155) 25698328

[53] Lane N, Allen JF, Martin W 2010 How did LUCA make a living? Chemiosmosis in the origin of life. Bioessays 32, 271–280. (10.1002/bies.200900131) 20108228

[54] Russell MJ, Hall AJ, Martin W 2010 Serpentinization as a source of energy at the origin of life. Geobiology 8, 355–371. (10.1111/j.1472-4669.2010.00249.x) 20572872

[55] Hoehler TM 2007 An energy balance concept for habitability. Astrobiology 7, 824–838. (10.1089/ast.2006.0095) 18163865

[56] German CR, Von Damm KL 2004 Hydrothermal processes. In Hrsg. treatise on geochemistry, volume 6: the oceans and marine geochemistry (eds Holland HD, Kurekian KK), pp. 181–222. London, UK: Elsevier.

[57] Amend JP, McCollom TM, Hentscher M, Bach W 2011 Catabolic and anabolic energy for chemolithoautotrophs in deep-sea hydrothermal systems hosted in different rock types. Geochim. Cosmochim. Acta 75, 5736–5748. (10.1016/j.gca.2011.07.041)

[58] Amend JP, Teske A 2005 Expanding frontiers in deep subsurface microbiology. Palaeogeogr. Palaeoclimatol. Palaeoecol. 219, 131–155. (10.1016/j.palaeo.2004.10.018)

[59] Brazelton WJ, Nelson B, Schrenk MO 2012 Metagenomic evidence for H-2 oxidation and H-2 production by serpentinite-hosted subsurface microbial communities. Front. Microbiol. 2, 268 (10.3389/fmicb.2011.00268) 22232619 PMC3252642

[60] Habicht KS, Gade M, Thamdrup B, Berg P, Canfield DE 2002 Calibration of sulfate levels in the Archean Ocean. Science 298, 2372–2374. (10.1126/science.1078265) 12493910

[61] Tarpgaard IH, Roy H, Jorgensen BB 2011 Concurrent low- and high-affinity sulfate reduction kinetics in marine sediment. Geochim. Cosmochim. Acta 75, 2997–3010. (10.1016/j.gca.2011.03.028)

[62] Nealson KH, Inagaki F, Takai K 2005 Hydrogen-driven subsurface lithoautotrophic microbial ecosystems (SLiMEs): do they exist and why should we care? Trends Microbiol. 13, 405–410. (10.1016/j.tim.2005.07.010) 16054814

[63] Weiss MC, Sousa FL, Mrnjavac N, Neukirchen S, Roettger M, Nelson-Sathi S, Martin WF 2017 The physiology and habitat of the last universal common ancestor. Nat. Microbiol. 1, 16116 (10.1038/nmicrobiol.2016.116) 27562259

[64] Cipolli F, Gambardella B, Marini L, Ottonello G, Vetuschi Zuccolini M 2004 Geochemistry of high-pH waters from serpentinites of the Gruppo di Voltri (Genova, Italy) and reaction path modeling of CO_2_ sequestration in serpentinite aquifers. Appl. Geochem. 19, 787–802. (10.1016/j.apgeochem.2003.10.007)

[65] Fritz P et al 1992 Deuterium and C-13 evidence for low-temperature production of hydrogen and methane in a highly alkaline groundwater environment in Oman Water-Rock Interaction, vols 1 and 2: vol. 1: Low Temperature Environments; vol. 2: Moderate and High Temperate Environments 793–796.

[66] Kelemen PB, Matter J, Streit EE, Rudge JF, Curry WB, Blusztajn J 2011 Rates and mechanisms of mineral carbonation in peridotite: natural processes and recipes for enhanced, *in situ* CO_2_ capture and storage. Annu. Rev. Earth Planet. Sci. 39, 545–576. (10.1146/annurev-earth-092010-152509)

[67] Lowell RP 2017 A fault-driven circulation model for the Lost City hydrothermal field. Geophys. Res. Lett. 44, 2703–2709. (10.1002/2016GL072326)

[68] Lang SQ, Fruh-Green GL, Bernasconi SM, Butterfield DA 2013 Sources of organic nitrogen at the serpentinite-hosted Lost City hydrothermal field. Geobiology 11, 154–169. (10.1111/gbi.12026) 23346942

[69] Reeves EP, McDermott JM, Seewald JS 2014 The origin of methanethiol in midocean ridge hydrothermal fluids. Proc. Natl Acad. Sci. USA 111, 5474–5479. (10.1073/pnas.1400643111) 24706901 PMC3992694

[70] McCollom TM, Seewald JS 2001 A reassessment of the potential for reduction of dissolved CO2 to hydrocarbons during serpentinization of olivine. Geochim. Cosmochim. Acta 65, 3769–3778. (10.1016/S0016-7037(01)00655-X)

[71] McCollom TM, Seewald JS 2003 Experimental constraints on the hydrothermal reactivity of organic acids and acid anions: I. Formic acid and formate. Geochim. Cosmochim. Acta 67, 3625–3644. (10.1016/S0016-7037(03)00136-4)

[72] McCollom TM, Seewald JS 2006 Carbon isotope composition of organic compounds produced by abiotic synthesis under hydrothermal conditions. Earth Planet. Sci. Lett. 243, 74–84. (10.1016/j.epsl.2006.01.027)

[73] Seewald JS, Zolotov MY, McCollom T 2006 Experimental investigation of single carbon compounds under hydrothermal conditions. Geochim. Cosmochim. Acta 70, 446–460. (10.1016/j.gca.2005.09.002)

[74] Jansen K, Thauer RK, Widdel F, Fuchs G 1984 Carbon assimilation pathways in sulfate reducing bacteria—formate, carbon dioxide, carbon monoxide, and acetate assimilation by desulfovibrio-baarsii. Arch. Microbiol. 138, 257–262. (10.1007/BF00402132)

[75] Penning H, Conrad R 2006 Carbon isotope effects associated with mixed-acid fermentation of saccharides by Clostridium papyrosolvens. Geochim. Cosmochim. Acta 70, 2283–2297. (10.1016/j.gca.2006.01.017)

[76] McDermott JM, Seewald JS, German CR, Sylva SP 2015 Pathways for abiotic organic synthesis at submarine hydrothermal fields. Proc. Natl Acad. Sci. USA 112, 7668–7672. (10.1073/pnas.1506295112) 26056279 PMC4485091

[77] Klein F, Grozeva NG, Seewald JS 2019 Abiotic methane synthesis and serpentinization in olivine-hosted fluid inclusions. Proc. Natl Acad. Sci. USA 116, 17 666–17 672. (10.1073/pnas.1907871116) 31427518 PMC6731755

[78] Miller HM, Chaudhry N, Conrad ME, Bill M, Kopf SH, Templeton AS 2018 Large carbon isotope variability during methanogenesis under alkaline conditions. Geochim. Cosmochim. Acta 237, 18–31. (10.1016/j.gca.2018.06.007)

[79] Delacour A, Fruh-Green GL, Bernasconi SM, Schaeffer P, Kelley DS 2008 Carbon geochemistry of serpentinites in the Lost City hydrothermal system (30° N, MAR). Geochimica Et Cosmochimica Acta 72, 3681–3702. (10.1016/j.gca.2008.04.039)

[80] Schwarzenbach EM, Lang SQ, Frah-Green GL, Lilley MD, Bernasconi SM, Mehay S 2013 Sources and cycling of carbon in continental, serpentinite-hosted alkaline springs in the Voltri Massif, Italy. Lithos 177, 226–244. (10.1016/j.lithos.2013.07.009)

[81] Klein F, Humphris SE, Guo WF, Schubotz F, Schwarzenbach EM, Orsi WD 2015 Fluid mixing and the deep biosphere of a fossil Lost City-type hydrothermal system at the Iberia Margin. Proc. Natl Acad. Sci. USA 112, 12 036–12 041. (10.1073/pnas.1504674112) PMC4593090 26324888

[82] Menez B, Pisapia C, Andreani M, Jamme F, Vanbellingen QP, Brunelle A, Richard L, Dumas P, Réfrégiers M 2018 Abiotic synthesis of amino acids in the recesses of the oceanic lithosphere. Nature 564, 59 (10.1038/s41586-018-0684-z) 30405236

[83] Pisapia C, Jamme F, Duponchel L, Ménez B 2018 Tracking hidden organic carbon in rocks using chemometrics and hyperspectral imaging. Sci. Rep. 8, 2396 (10.1038/s41598-018-20890-4) 29402966 PMC5799262

[84] Pisapia C et al 2017 Mineralizing filamentous bacteria from the Prony Bay hydrothermal field give new insights into the functioning of serpentinization-based subseafloor ecosystems. Front. Microbiol. 8, 57 (10.3389/fmicb.2017.00057) 28197130 PMC5281578

[85] Schrenk MO, Brazelton WJ, Lang SQ. 2013 Serpentinization, carbon, and deep Life. Rev. Mineral. Geochem. 75, 575–606. (10.2138/rmg.2013.75.18)

[86] Sorokin DY, Tourova TP, Mussmann M, Muyzer G 2008 *Dethiobacter alkaliphilus* gen. nov. sp. nov., and *Desulfurivibrio alkaliphilus* gen. nov. sp. nov.: two novel representatives of reductive sulfur cycle from soda lakes. Extremophiles 12, 431–439. (10.1007/s00792-008-0148-8) 18317684

[87] Knapp AN, DiFiore PJ, Deutsch C, Sigman DM, Lipschultz F 2008 Nitrate isotopic composition between Bermuda and Puerto Rico: implications for N_2_ fixation in the Atlantic Ocean. Global Biogeochem. Cycles 22, 1–14. (10.1029/2007GB003107)

[88] Marconi D, Weigand MA, Rafter PA, Mcilvin MR, Forbes M, Casciotti KL, Sigman DM 2015 Nitrate isotope distributions on the US GEOTRACES North Atlantic cross-basin section: signals of polar nitrate sources and low latitude nitrogen cycling. Mar. Chem. 177, 143–156. (10.1016/j.marchem.2015.06.007)

[89] Bourbonnais A, Lehmann MF, Butterfield DA, Juniper SK 2012 Subseafloor nitrogen transformations in diffuse hydrothermal vent fluids of the Juan de Fuca Ridge evidenced by the isotopic composition of nitrate and ammonium. Geochem. Geophys. Geosyst. 13, 1–23. (10.1029/2011GC003863)

[90] Lee S, Fuhrman JA. 1987 Relationships between biovolume and biomass of naturally derived marine bacterioplankton. Appl. Environ. Microbiol. 53, 1298–1303. 16347362 10.1128/aem.53.6.1298-1303.1987 PMC203858

[91] Fagerbakke KM, Heldal M, Norland S 1996 Content of carbon, nitrogen, oxygen, sulfur and phosphorus in native aquatic and cultured bacteria. Aquat. Microbial Ecol. 10, 15–27. (10.3354/ame010015)

[92] Gundersen K, Heldal M, Norland S, Purdie DA, Knap AH 2002 Elemental C, N, and P cell content of individual bacteria collected at the Bermuda Atlantic Time-Series Study (BATS) site. Limnol. Oceanogr. 47, 1525–1530. (10.4319/lo.2002.47.5.1525)

[93] Kandler O, Hippe H 1977 Lack of peptidoglycan in cell walls of methanosarcina barkeri. Arch. Microbiol. 113, 57–60. (10.1007/BF00428580) 889387

[94] Minagawa M, Wada E 1986 Nitrogen isotope ratios of red tide organisms in the East China Sea—a characterization of biological nitrogen fixation. Mar. Chem. 19, 245–259. (10.1016/0304-4203(86)90026-5)

[95] Carpenter EJ, Harvey HR, Fry B, Capone DG 1997 Biogeochemical tracers of the marine cyanobacterium Trichodesmium. Deep Sea Res. Part I Oceanogr. Res. Papers 44, 27–38. (10.1016/S0967-0637(96)00091-X)

[96] Javoy M, Pineau F 1991 The volatiles record of a popping rock from the Mid-Atlantic Ridge at 14°N chemical and isotopic composition of gas trapped in the vesicles. Earth Planet. Sci. Lett. 107, 598–611. (10.1016/0012-821X(91)90104-P)

[97] Marty B, Humbert F 1997 Nitrogen and argon isotopes in oceanic basalts. Earth Planet. Sci. Lett. 152, 101–112. (10.1016/S0012-821X(97)00153-2)

[98] Mehta MP 2006 Biological nitrogen fixation in deep-sea and hydrothermal vent environments. Seattle, WA: University of Washington.

[99] Page L, Hattori K, Guillot S 2018 Mantle wedge serpentinites: a transient reservoir of halogens, boron, and nitrogen for the deeper mantle. Geology 46, 883–886. (10.1130/G45204.1)

[100] Hooper DU, Vitousek PM 1998 Effects of plant composition and diversity on nutrient cycling. Ecol. Monogr. 68, 121–149. (10.1890/0012-9615(1998)068[0121:EOPCAD]2.0.CO;2)

[101] Wheat CG, McManus J, Mottl MJ, Giambalvo E, 2003 Oceanic phosphorus imbalance: magnitude of the mid-ocean ridge flank hydrothermal sink. Geophys. Res. Lett. 30, 1–4. (10.1029/2003GL017318)

[102] Wheat CG, Feely RA, Mottl MJ 1996 Phosphate removal by oceanic hydrothermal processes: an update of the phosphorus budget in the oceans. Geochim. Cosmochim. Acta 60, 3593–3608. (10.1016/0016-7037(96)00189-5)

[103] Bradley AS, Fredricks H, Hinrichs K-U, Summons RE 2009 Structural diversity of diether lipids in carbonate chimneys at the Lost City hydrothermal field. Org. Geochem. 40, 1169–1178. (10.1016/j.orggeochem.2009.09.004)

[104] Nisbet EG, Sleep NH 2001 The habitat and nature of early life. Nature 409, 1083–1091. (10.1038/35059210) 11234022

[105] Holm NG, Dumont M, Ivarsson M, Konn C 2006 Alkaline fluid circulation in ultramafic rocks and formation of nucleotide constituents: a hypothesis. Geochem. Trans. 7, 1–7. (10.1186/1467-4866-7-7) PMC1550712 16867193

[106] Liebmann J, Schwarzenbach EM, Fruh-Green GL, Boschi C, Rouméjon S, Strauss H, Wiechert U, John T 2018 Tracking water-rock interaction at the Atlantis Massif (MAR, 30°N) using sulfur geochemistry. Geochem. Geophys. Geosyst. 19, 4561–4583. (10.1029/2018GC007813)

[107] Alt JC, Shanks WC, Bach W, Paulick H, Garrido CJ, Beaudoin G 2007 Hydrothermal alteration and microbial sulfate reduction in peridotite and gabbro exposed by detachment faulting at the Mid-Atlantic Ridge, 15°20‘N (ODP Leg 209): a sulfur and oxygen isotope study. Geochem. Geophys. Geosyst. 8, 22 (10.1029/2007GC001617)

[108] Holm RH, Kennepohl P, Solomon EI 1996 Structural and functional aspects of metal sites in biology. Chem. Rev. 96, 2239–2314. (10.1021/cr9500390) 11848828

[109] Glass JB, Orphan VJ 2012 Trace metal requirements for microbial enzymes involved in the production and consumption of methane and nitrous oxide. Front. Microbiol. 3, 61 (10.3389/fmicb.2012.00061) 22363333 PMC3282944

[110] Zerkle AL, House CH, Brantley SL 2005 Biogeochemical signatures through time as inferred from whole microbial genomes. Am. J. Sci. 305, 467–502. (10.2475/ajs.305.6-8.467)

[111] Michalski JR, Onstott TC, Mojzsis SJ, Mustard J, Chan QHS, Niles PB, Johnson SS 2018 The Martian subsurface as a potential window into the origin of life. Nat. Geosci. 11, 21 (10.1038/s41561-017-0015-2)

[112] Allen DE, Seyfried WE 2004 Serpentinization and heat generation: constraints from Lost City and rainbow hydrothermal systems. Geochim. Cosmochim. Acta 68, 1347–1354. (10.1016/j.gca.2003.09.003)

[113] Hand KP, Carlson RW, Chyba CF 2007 Energy, chemical disequilibrium, and geological constraints on Europa. Astrobiology 7, 1006–1022. (10.1089/ast.2007.0156) 18163875

[114] Cabrol NA 2018 The coevolution of life and environment on Mars: an ecosystem perspective on the robotic exploration of biosignatures. Astrobiology 18, 1–27. (10.1089/ast.2017.1756) 29252008 PMC5779243

